# Collagen-dependent platelet dysfunction and its relevance to either mitochondrial ROS or cytosolic superoxide generation: a question about the quality and functional competence of long-stored platelets

**DOI:** 10.1186/s12959-020-00233-y

**Published:** 2020-08-31

**Authors:** Ehteramolsadat Hosseini, Saba Hojjati, Safoora Afzalniaye gashti, Mehran Ghasemzadeh

**Affiliations:** Blood Transfusion Research Centre, High Institute for Research and Education in Transfusion Medicine, Iranian Blood Transfusion Organization Building, Hemmat Exp Way, Next to the Milad Tower, Tehran, Iran

**Keywords:** Adhesion, Aggregation, Collagen, Platelet, Reactive oxygen species, Spreading, Superoxide, Thrombosis, Transfusion

## Abstract

**Background:**

Upon vascular damage, the exposed subendothelial matrix recruits circulating platelets to site of injury while inducing their firm adhesion mainly via GPVI-collagen interaction. GPVI also supports aggregatory and pro-coagulant functions in arterial shear rate even on the matrix other than collagen. Reactive oxygen species (ROS) modulate these stages of thrombosis; however augmented oxidant stress also disturbs platelet functions. Stored-dependent platelet lesion is associated with the increasing levels of ROS. Whether ROS accumulation is also relevant to collagen-dependent platelet dysfunction is the main interest of this study.

**Methods:**

Fresh PRP-PCs (platelet concentrates) were either stimulated with potent ROS-inducers PMA and CCCP or stored for 5 days. Intra-platelet superoxide (O_2_^−−^) or mitochondrial-ROS and GPVI expression were detected by flowcytometery. GPVI shedding, platelet aggregation and spreading/adhesion to collagen were analyzed by western blot, aggregometry and fluorescence-microscopy, respectively**.**

**Results:**

Mitochondrial-ROS levels in 5 days-stored PCs were comparable to those induced by mitochondrial uncoupler, CCCP while O_2_^−−^ generations were higher than those achieved by PMA. Shedding levels in 5 days-stored PCs were higher than those induced by these potent stimuli. GPVI expressions were reduced comparably in CCCP treated and 5 days-stored PCs. Platelet adhesion was also diminished during storage while demonstrating significant reverse correlation with GPVI shedding. However, only firm adhesion (indicated by platelets spreading or adhesion surface area) was relevant to GPVI expression. Platelet adhesion and aggregation also showed reverse correlations with both O2^−−^ and mitochondrial-ROS formations; nonetheless mitochondrial-ROS was only relevant to firm adhesion.

**Conclusion:**

As a sensitive indicator of platelet activation, GPVI shedding was correlated with either simple adhesion or spreading to collagen, while GPVI expression was only relevant to platelet spreading. Thereby, if the aim of GPVI evaluation is to examine platelet firm adhesion, expression seems to be a more specific choice. Furthermore, the comparable levels of ROS generation in 5 days-stored PCs and CCCP treated platelets, indicated that these products are significantly affected by oxidative stress. Reverse correlation of accumulating ROS with collagen-dependent platelet dysfunction is also a striking sign of an oxidant-induced lesion that may raise serious question about the post-transfusion quality and competence of longer-stored platelet products.

## Research highlights

- Platelet storage is associated with increasing levels of ROS and GPVI shedding, versus decreeing levels of GPVI expression.
- 5 days-stored platelets show oxidative stress comparable to those induced by potent stimuli.
- 5 days-stored platelets show GPVI shedding further than those induced by potent stimuli, PMA or CCCP.
- GPVI expressions are reduced comparably in CCCP treated and 5 days-stored PCs.
- Platelet spreading on collagen is relevant to the increasing levels of ROS and GPVI shedding as well as its reducing expression during storage.
- Collagen-dependent platelet aggregation is reversely correlated with ROS accumulation in stored PCs.
- Oxidant stress raises questions about the functional competence of 5 days-stored platelets.

## Background

Up on vascular damage, platelets adhesion and spreading to the site of injury is considered as the first and crucial stage of thrombus formation and hemostatic events to control the hemorrhage. Spread platelets not only cover the vasculature breaches but they also provide an efficient scaffold which engages other free flowing platelets to establish primary aggregates developing to stable thrombi which effectively seal endothelial damage and stop the hemorrhage. Followed by vascular lesion, the exposure of sub-endothelial immobilized vWF/collagen recruits platelets to the injury site. Classically, the interaction between immobilized vWF and GPIbα tethers platelets to injured blood vessels while slowing platelets down to better interact with sub-endothelial matrix especially under higher shear force condition. However, notably this is the engagement of platelet GPVI receptor and collagen that mainly supports platelet firm adhesion and spreading to the site of vascular injury [1, 2]. GPVI ligation to collagen also induces potent inside out signals which play important roles in the enhancement of integrin activation leading to platelet aggregation, granule release and pro-coagulant function [3, 4]. It is postulated that patients with GPVI deficiency can suffer from prolonged bleeding [5, 6] and this is in addition to the emerged critical role of this receptor in the regulation of atherothrombosis. Several line of evidences showed that the inhibition of platelet GPVI with specific antibodies or antagonizing its binding to immobilized collagen through soluble dimeric GPVI attenuates arterial thrombosis whereas not compromising physiological hemostasis [7]. Most recently, Nagy et.al indicated that GPVI can promote platelet aggregation and PS exposure in arterial shear rate even on the matrix other than collagen [8]. Given the clinical importance of GPVI in hemostasis, the efficient function of this receptor and its binding capacities to collagen during storage can be of interest for whom concern about the efficacy and quality of therapeutic platelets which affected by platelet storage lesion (PSL). Studies have shown that besides many indicators of PSL, the gradual loss of platelet adhesiveness to different reactive matrixes such as collagen may be also considered as other markers that indicate platelet increasing dysfunction during storage [9, 10]. Previous studies that highlighted the prominent shedding of GPVI receptors in stored platelets also indicated its reverse correlation with platelet adhesive capacity to collagen [11]. It has been generally shown that upon platelet activation, ADAM-dependent shedding of platelet adhesion receptors can be modulated by Ca^2+^ elevation, protein kinase C (PKC) activation, PS exposure and caspase activity [1, 12]. Alternatively, platelet activation and storage are also associated with the generation of reactive oxygen species (ROS) which act as signaling molecules modulating different aspects of platelet functions [13, 14]. Several enzymatic pathways including NADPH oxidase, xanthine oxidase, PI3 kinase and protein kinase C contribute to ROS generation by blood platelets of which NADPH oxidase (NOX1/2) has major role [15]. NOX activation elicits the generation of O_2_^−−^, an unstable product which is rapidly converted to H_2_O_2_ by the act of superoxide-dismutase (SOD). Mitochondrial oxidative metabolisms are also considered as another source for ROS while mitochondrial-originated ROS may amplify their generation by NOX activation or vice versa [16]. ROS can be involved in platelet adhesion receptors shedding which may reduce their expression, affecting platelet adhesive function [17]. Up on platelet activation and adhesion, calcium elevation is associated with increased levels of intracellular ROS which oxidize cysteine residues located on cysteine-rich domain of ADAMs directly activating these proteolytic molecules to shed adhesion receptors. On the other hand, the interaction of ROS with intracellular cytoplasmic domains of ADAMs may also increase their affinity with substrate [1, 18]. In addition, direct and indirect interactions of ROS with cytoplasmic domain of GPVI can also modulate its shedding events. In a direct pathway, GPVI ligation induces rapid oxidation of an unpaired thiol in the cytoplasmic tail of receptor leading to GPVI dimerization and its ectodomain metalloproteolysis [19, 20] while indirectly, the oxidation of cysteine residues of different related kinases including p38-MAPK (mitogen-activated protein kinase), PKC (protein kinase C) and PI3K can also be involved in shedding events [21–23]. In clinical bases, some studies found direct correlations between oxidative stress and bleeding complications in patients. The most current study has been conducted in patients undergoing continuous-flow left ventricular assist device (CF-LVAD) implantation who experienced higher levels of GPVI shedding correlated with the elevated markers of oxidative stress [24]. So far, several studies indicated that increasing levels of ROS generation in stored platelets is associated with PSL. Most recently, we have indicated that in stored platelets, either the ROS scavenging or the reduction of their generation by NOX inhibition, can effectively promote platelet viability while reducing PSL effect during storage [25]. Now, considering the role of oxidative stress in the modulation of GPVI, here we also investigated whether increasing levels of ROS during platelet storage can be functionally relevant to the storage-dependent loss of platelet spreading/ adhesion to collagen.

## Method and materials

### Sample preparation

6 Platelet rich plasma-platelet concentrates (PRP-PCs) were produced from the whole blood bags (with CPD anti-coagulant) donated by volunteers under IBTO’s regulations. Each bag contained 60–70 ml PCs with more than 1 × 10^9^ platelets/ml. To perform the relevant assays, on the day 0 of storage (at the most 3 h after the platelet preparation including resting time), 5 mL of freshly prepared platelets were taken from each bag. Sample collection was performed under sterile condition as previously described [11]. PCs were then kept in shaker incubator at 20–24 °C (circular agitator) till the next sampling stage on day 5 of storage. For each sample adding a designated amount of Tyrode buffer (10 mM Hepes, 12 mM NaHCO3, 137 mM NaCl, 2.7 mM KCl, 5 mM glucose, 1 mM CaCl2; pH = 7.4), platelets were washed and isolated as also described previously [26]. For flowcytometry and adhesion analysis, platelet counts were adjusted to 2 × 10^7^/ml. Platelet poor plasma (PPP) obtained from PRP with the platelet count of 5 × 10^8^/ml was subjected to two steps ultracentrifugation (2 × 10^4^ g for 30 min each time) and microparticles (MPs)-free supernatant was separated and kept in − 20 °C to be analyzed by western blotting for the evaluation of shed proteins. The study was approved by the local ethical committee and the informed consent was obtained from the blood donors by Iranian Blood Transfusion Organization (IBTO).

### QC parameters of PCs

See supplementary Method.

### Analysis of intra-platelet ROS generation

Dihydrorhodamine (DHR) 123 was already reported to be used for evaluation of mitochondrial ROS production in different cell lines including platelets [27–32]. In this study, DHR123 was applied for the evaluation of mitochondrial ROS production. To analyze cytosolic production of O_2_^−−^, dihydroethidium (DHE) has also been used. See supplementary Method and figure for further details (sup Fig. 1).

### Western blot analysis to evaluate GPVI shedding

See supplementary Method.

### Flow cytometery analysis to detect GPVI expression

See supplementary Method.

### Platelet adhesion to collagen matrix

Glass coverslips were incubated with 100 μg/ml collagen type I in PBS for 1 h at room temperature and then followed by washing steps, those coverslips were incubated again with 2% bovine serum albumin (as blocking solution) for 30 min at room temperature. Excess solution was removed by three washes with Tyrode’s buffer and coverslips were kept immersed in Tyrode’s buffer until required. Human platelets (2 × 10^7^/ml) were then allowed to adhere on coverslips for 30 min at 37 °C under mild stirring condition. Non-adherent platelets were aspirated and adherent platelets fixed with 3.7% formaldehyde for 15 min. Adherent platelets were visualized by fluorescence microscope (100x objectives). For this purpose, prior to each experiment platelets were labeled with fluorescence dye, DIOC6 and then platelets were subjected to adhesion assays. Total number of adhered platelets and of those the percentages of spread platelets were calculated. The total surface area covered by platelets (μm^2^) was also quantified using ImageJ software by ImageJ software (Research Services Branch, National Institute of Mental Health, Bethesda, MD, USA). Therefore, in this study, we have got three key parameters to address platelet adhesion. The first one is a general parameter described as the “*number of adhered platelets*” regardless of the fact that whether they just simply adhered or also spread over the matrix. The second parameter is “*the percentage of platelet spreading*” which only includes the platelets that fully spread over the matrix and the last one is “*platelet adhesion area*” which is calculated by the total surface area of the matrix covered with all platelets.

### Platelet aggregation

Aggregation tests were conducted by a Lumi-Aggregometers (CHRONO-LOG 700-X, USA). For this purpose, the aggregometer was calibrated to 100 and 0% light transmittance with the PPP and PRP, respectively. Designated PRP samples in presence of collagen (5 μg/mL) were then subjected to aggregometer while allowing the aggregation pattern to be generated for 10 min under stirring condition.

## Results

### Exaggerated intra-platelet ROS generation in 5 days-stored PCs

**Fig. 1:**
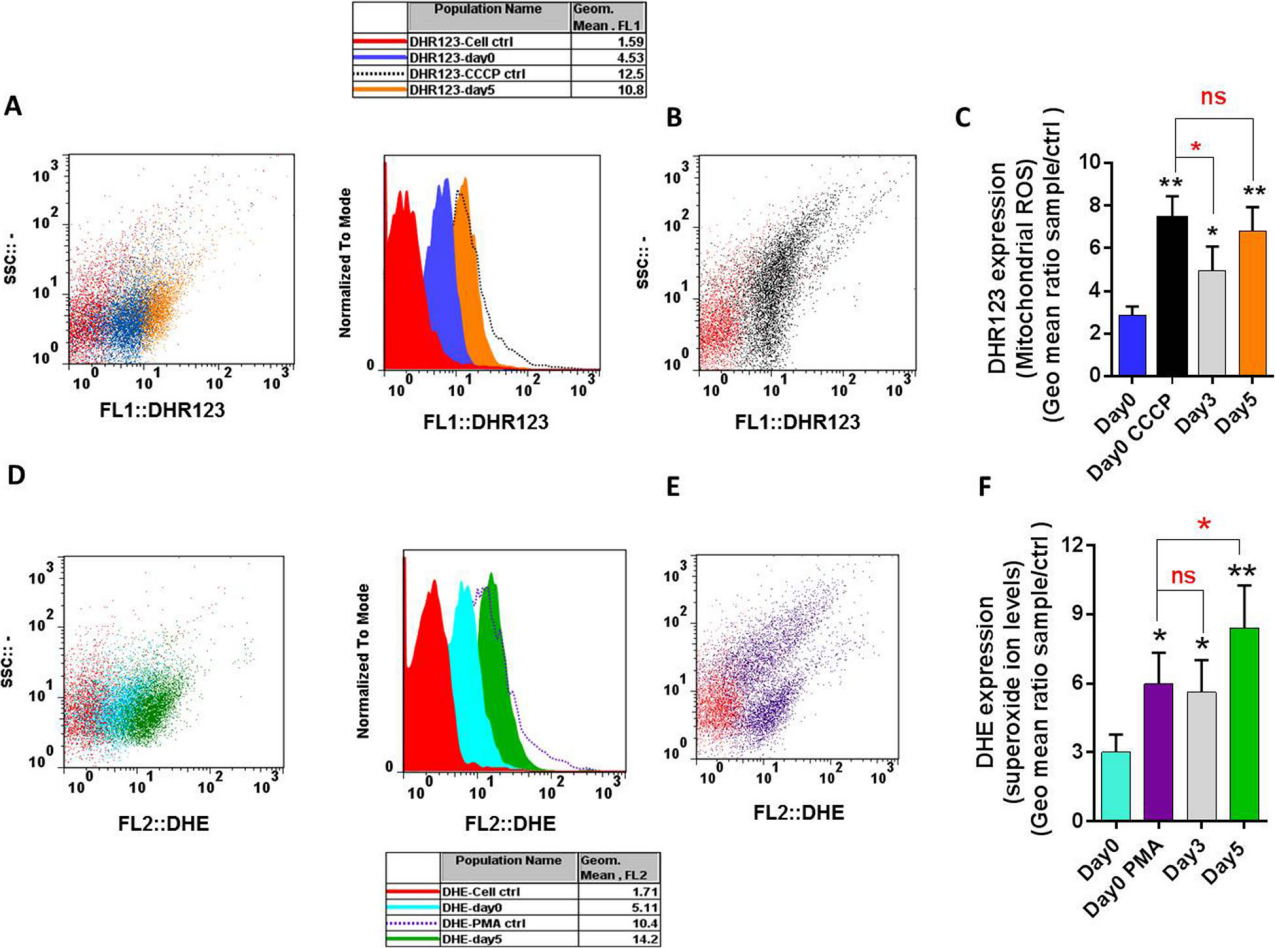
Exaggerated Intra-platelet ROS generation in 5 days-stored PCs. **a**, **b**, **d** and **e** show the representative histograms and dot plots illustrating the expressions of DHR123 and DHE respectively detected in FL1 and FL2. Graph (**c**) shows significant increase of mitochondrial ROS in both CCCP induced (*p* < 0.01) and 5-days stored platelets (*p* < 0.01; analyzed by Kruskal-Wallis test with Dunn’s multiple comparison) with not significant (ns) difference between them (assessed by Mann–Whitney U test). Graph (**f**) shows significant increase of Superoxide in both PMA induced (*p* = 0.045) and 5-days stored platelets (*p* < 0.05; analyzed by Kruskal-Wallis test with Dunn’s multiple comparison) with significant (*p* = 0.048) difference between them (assessed by Mann–Whitney U test). Note: PCs; platelets concentrated, **p < 0.01, *p < 0.05, ns: not significant *p* > 0.05 (*n* = 6)

Non-physiological agents including PMA and CCCP induce intra-platelet ROS generation from different sources. For this study, CCCP has been used as a typical mitochondrial uncoupler that disrupts oxidative phosphorylation inducing mitochondrial dysfunction and augmented ROS generation which here detected in a DHR123- dependent assay. On other hand, we alternatively treated fresh platelets (obtained from 0 day-stored PCs) with PMA to induce NOX activity with the highest levels of cytosolic ROS detected by DHE assay. As shown in Fig. 1a & c, treatment of 0 day-stored platelets (fresh platelets) with CCCP increased ROS to ~ 2.5 folds higher than that observed in non-treated one. In addition, platelets incubation with PMA elevated O_2_^−−^ to levels that were two times more than those observed in non-treated 0 day-stored platelets (Fig. 1e & f). Previous studies showed increasing levels of ROS generation in platelets during storage with the highest levels of ROS accumulation in 5-day stored platelets. However, the significance of this elevation was under question. As showed in Fig. 1c, the levels of mitochondrial ROS accumulated in 5-day stored platelets were comparable to that induced by CCCP. Interestingly, O_2_^−−^ generation in 5-day stored platelets was significantly (*p* < 0.05) higher than that achieved by PMA while the levels of O_2_^−−^ formed in 3-day stored platelets was comparable to those observed in PMA stimulated fresh platelets (Fig. 1f).

### GPVI shedding in response to non-physiological stimuli versus storage

**Fig. 2:**
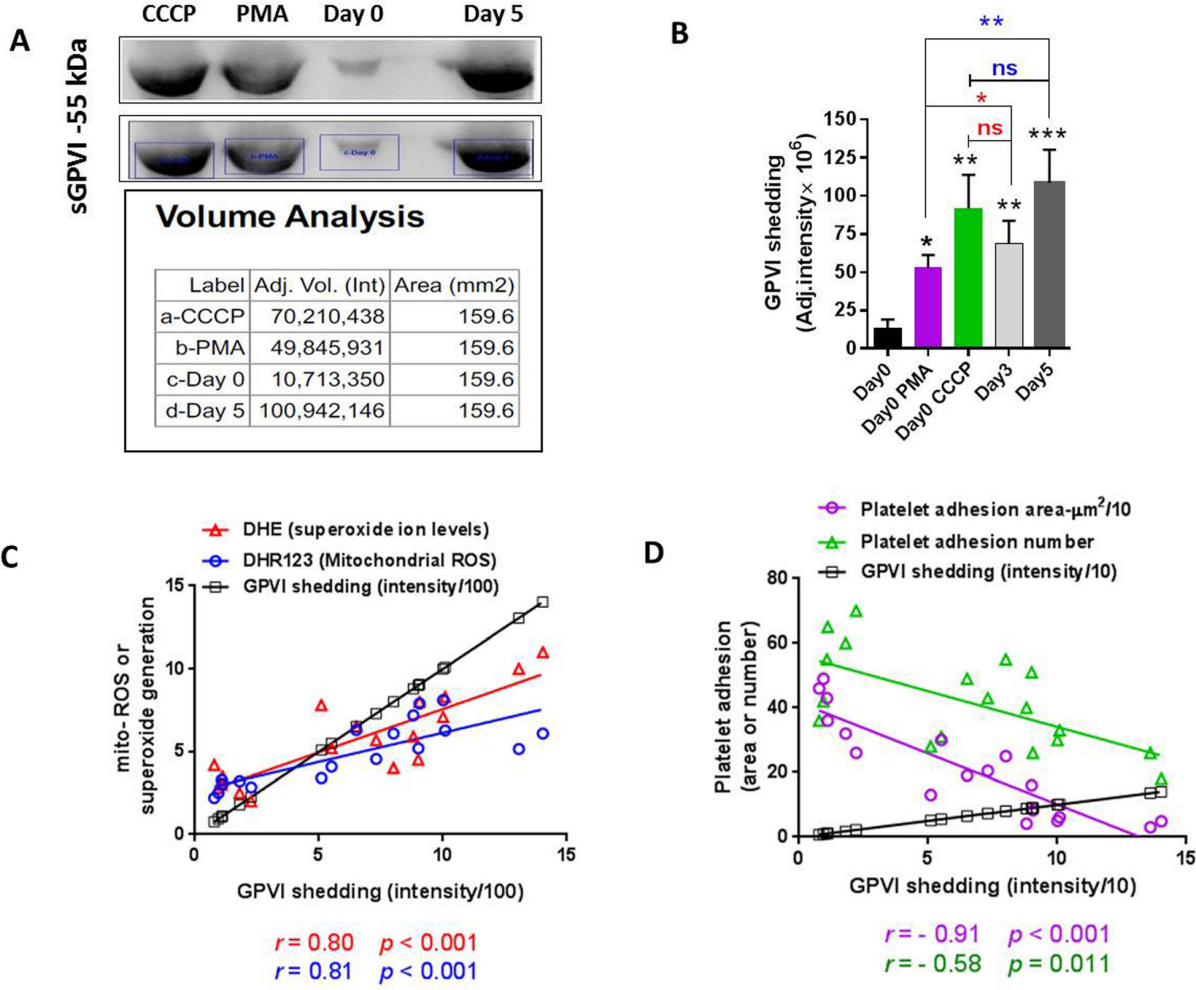
GPVI shedding and its correlation with adhesion capacities and ROS generation in stored platelets. **a** shows representative western blot image illustrating shedding patterns of GPVI in response to non-physiological stimuli PMA or CCCP versus 5 days storage. As demonstrated in graph (**b**) either PMA- (*p* < 0.05) or CCCP-induced (*p* < 0.01) platelets (from 0 day-stored PCs) and 5 days-stored PCs (*p* < 0.001) showed significantly higher levels of shedding than that of 0 day-stored PCs (analyzed by Kruskal-Wallis test with Dunn’s multiple comparison). There was no significant difference between shedding levels of CCCP- induced and 5 days-stored platelets (assessed by Mann–Whitney U test). Graph (**c**) shows direct correlations between GPVI shedding and either mitochondrial ROS (*r =* 0.81; *p* < 0.001) or cytosolic superoxide generation (*r* = 0.80; *p* < 0.001). A prominent reverse correlation were observed between GPVI shedding and platelet adhesion area (*r* = − 0.91; *p <* 0.001), which is more potent than that observed for simple adhesion number (*r* = − 0.58; *p* = 0.01) (Graph **d**). Note: Correlations were analyzed by Spearman’s rank correlation test. *P* values of less than 0.05 were considered to be significant. Graphs have been plotted using accumulating data obtained during storage from day 0 to day 5. ***p < 0.001, **p < 0.01, *p < 0.05, ns: not significant *p* > 0.05 (*n* = 6)

Platelet storage showed to be associated with the increasing levels of GPVI shedding with the highest levels observed in 5 days-stored PCs [11]. Fig. 2a presents a blot image illustrating the highest levels of GPVI shedding in 5 days-stored platelet with that of day 0 which is at the lowest levels ever tested here. Figure 2a & b also showed the significant increments of GPVI shedding levels in response to CCCP (100 μM) and PMA (10 μM) in 0 day-stored platelets (freshly prepared PCs). The treatment of platelet with the CCCP indicated to induce higher levels of GPVI shedding compared to that obtained by PMA. Of note, the increased levels of shedding induced by PMA are still lower than those observed in 3 or 5 days-stored PCs in which the shedding levels are comparable to those induced by CCCP. With further evaluation (Fig. 2c), a direct correlation was found between GPVI shedding and either mitochondrial ROS (*r* = 0.81; *p* < 0.001) or cytosolic superoxide generation (*r* = 0.8; *p* < 0.001).

### GPVI expression in response to non-physiological stimuli versus storage

**Fig. 3:**
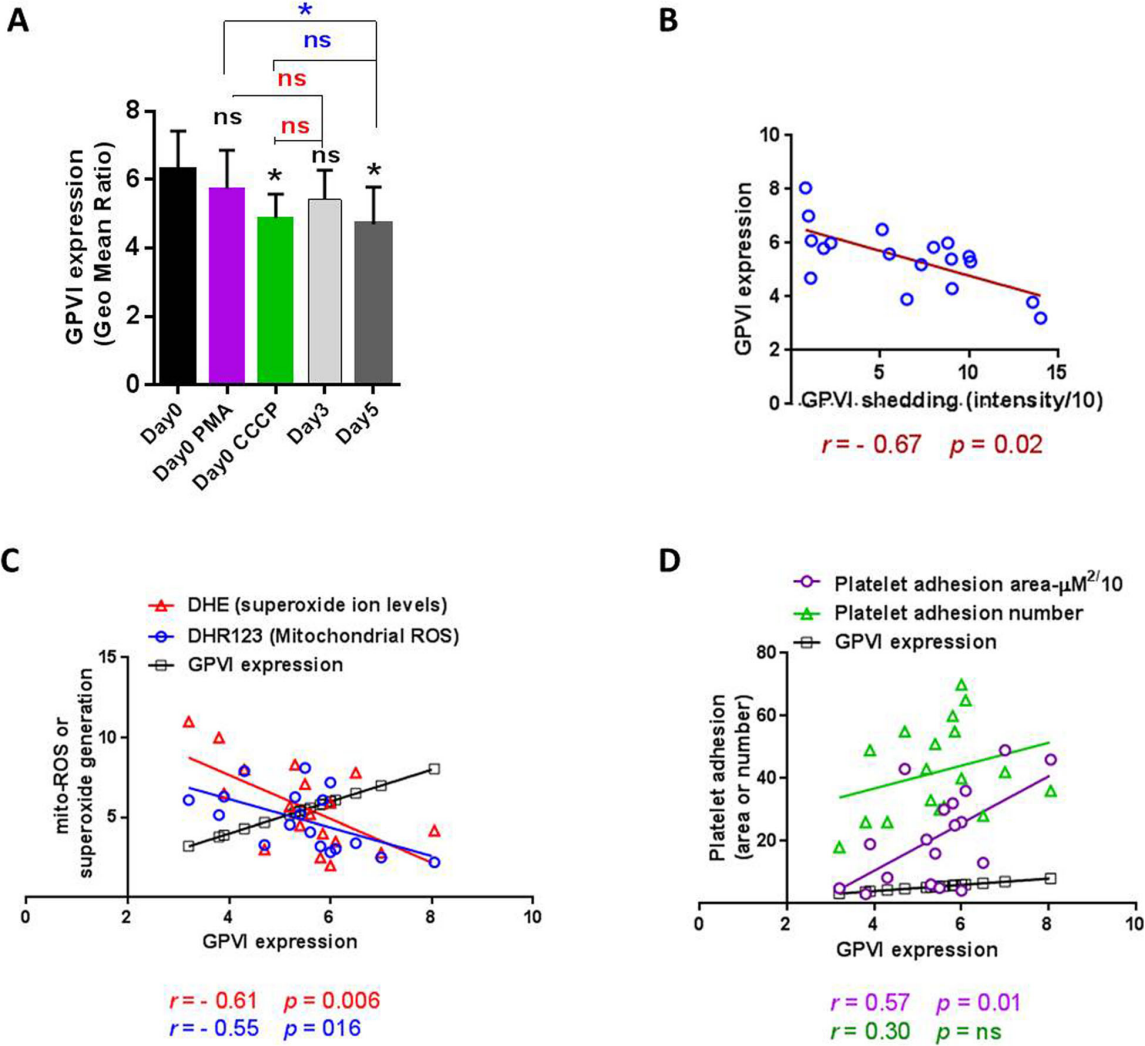
GPVI expression and its correlation with adhesion capacities and ROS generation in stored platelets. As demonstrated in graph (**a**), CCCP-induced (*p <* 0.05) platelets (from 0 day-stored PCs) and 5 days-stored PCs (*p <* 0.05) showed significantly lower levels of GPVI expression than that of 0 day-stored PCs (analyzed by Kruskal-Wallis test with Dunn’s multiple comparison). No significant difference was shown between expression levels of CCCP- induced and 5 days-stored platelets (assessed by Mann–Whitney U test). Graph (**b**) shows reverse correlation between GPVI shedding and expression (*r =* − 0.67; *p* = 0.02). Reverse correlations between GPVI expression and either mitochondrial ROS (*r =* − 0.55; *p* = 0.016) or cytosolic superoxide generation (*r* = − 0.61; *p* < 0.006) have been observed in graph (**c**). A direct correlation was showed between GPVI expression and platelet adhesion area (*r =* 0.57; *p* = 0. 01), whereas no correlation was detected between GPVI expression and simple adhesion number (graph **d**). Note: Correlations were analyzed by Spearman’s rank correlation test. *P* values of less than 0.05 were considered to be significant. Correlation graphs have been plotted using accumulating data obtained during storage from day 0 to day 5. *p < 0.05, ns: not significant *p* > 0.05 (*n* = 6)

Results showed decreasing levels of GPVI expression during storage with the significant differences starting from day 5 (*p* < 0.05), which its reduction was comparable to that observed in CCCP treated platelets. However, PMA did not appear to reduce GPVI expression (Fig. 3a). As shown in Fig. 3b there was a significant reverse correlation between GPVI shedding and its expression (*r* = − 0.67; *p* = 0.02). Further evaluations (Fig. 3c) also indicated a significant reverse correlation between GPVI expression and either mitochondrial ROS (*r* = − 0.61; *p* = 0.006) or cytosolic superoxide generation (*r* = − 0.55; *p* = 0.016).

### Platelet spreading on collagen in stored platelets

**Fig. 4:**
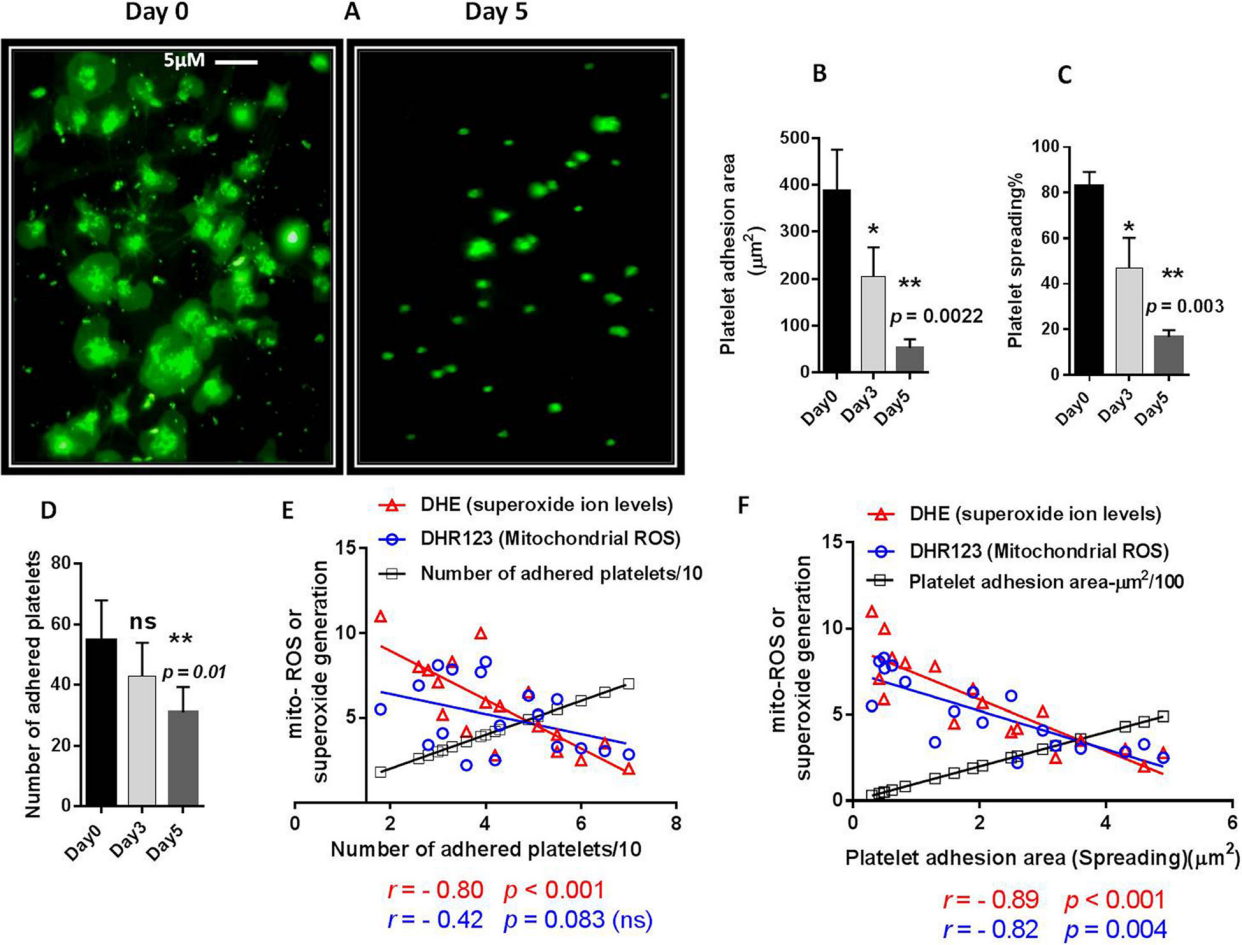
Platelets spreading/adhesion on collagen and its correlation with ROS generation in stored platelets. **a** presents a demonstrative image depicting platelets spreading capacities which seriously attenuated in 5-day stored PCs. Graphs **b** & **c** show significant reduction of platelet spreading and adhesion surface area in 3 and 5 days-stored PCs compared to fresh one (0 day-stored PCs). Graph **d** shows the number of adhered platelets in both 3 and 5 days-stored PCs with the significant reduction observed for day 5 of storage. (**e**), shows no correlation between mitochondrial ROS and simple adhesion of platelets to collagen (*r* = − 0.42; *p* = ns). However, this adhesive pattern showed to be reversely relevant to platelet superoxide generation (*r* = − 0.8; *p* < 0.001). As shown in (**f**), platelet spreading (platelet adhesion area) is significantly correlated with either mitochondrial ROS (*r* = − 0.82; *p* = 0.004) or cytosolic superoxide generation (*r* = − 0.89; *p* < 0.001). Note: Correlations were analyzed by Spearman’s rank correlation test. P values of less than 0.05 were considered to be significant. Correlation graphs have been plotted using accumulating data obtained during storage from day 0 to day 5. ***p < 0.001, **p < 0.01, *p < 0.05, ns: not significant *p* > 0.05 (*n =* 6)

In a dynamic process platelet adhesion to collagen is followed by spreading process which reflects platelet metabolic and functional abilities. Previous studies showed decreeing adhesion capacity of platelet during storage; however there were no data to evaluate platelets spreading to collagen as an important functional marker of storage-dependent lesion. Figure 4a presents a demonstrative image depicting fresh platelets spreading capacities which seriously attenuated in 5-day stored PCs. Graph 4B demonstrates tremendous reduction in platelet adhesion surface area(*p* = 0.0022) in 5 days-stored platelets compared to fresh one (one day-stored). Of note, the percentage of spread platelets (*p* = 0.003) and even with less significance (*p* = 0.01) the number of adhered platelets also decreased in 5 days-stored platelets (Fig. 4 c & d). However, as showed in figures the significant reductions in platelet spreading and adhesion surface area have been actually started from day 3 of storage, while the numbers of adhered platelets have not shown significant changes.

### The correlation of platelet GPVI expression/shedding with adhesion capacities in stored platelets

GPVI is a main receptor involved in platelet firm adhesion and spreading to the site of vascular injury [33]. We already showed the significant reverse correlation between platelet simple adhesion to collagen matrix and GPVI shedding [11]. Here, in addition to simple adhesion we also evaluated the correlation between platelet spreading to collagen (platelet adhesion area) and GPVI shedding in stored PCs. As shown in Fig. 2d, a prominent reverse correlation between GPVI shedding and platelet adhesion area was observed here, which according to its correlation indexes (*r* = − 0.91; *p* < 0.001) is more potent than that observed for simple adhesion (*r* = − 0.58; *p* = 0.011). Unlike GPVI shedding, its expression was not relevant to platelet adhesion number (simple adhesion). However, GPVI expression showed significant direct correlation (*r =* 0.57; *p =* 0.01) with platelet firm adhesion to collagen which presented as platelet adhesion area during storage (Fig. 3d).

### The correlation of platelet spreading (platelet adhesion area) on collagen with either mitochondrial ROS or cytosolic superoxide generation in stored platelets

As showed in Fig. 4e, whereas DHR123 as an indicator of mitochondrial ROS was not significantly correlated with the simple adhesion of platelets to collagen, this adhesive pattern showed to be reversely relevant to platelet superoxide generation detected by DHE expression (*r* = − 0.80; *p* < 0.001). However, platelet spreading showed to be significantly correlated with either mitochondrial ROS (*r* = − 0.82; *p* = 0.004) or cytosolic superoxide generation (*r* = − 0.89; *p* < 0.001) (Fig. 4f).

### Collagen-induced platelet aggregation

**Fig. 5:**
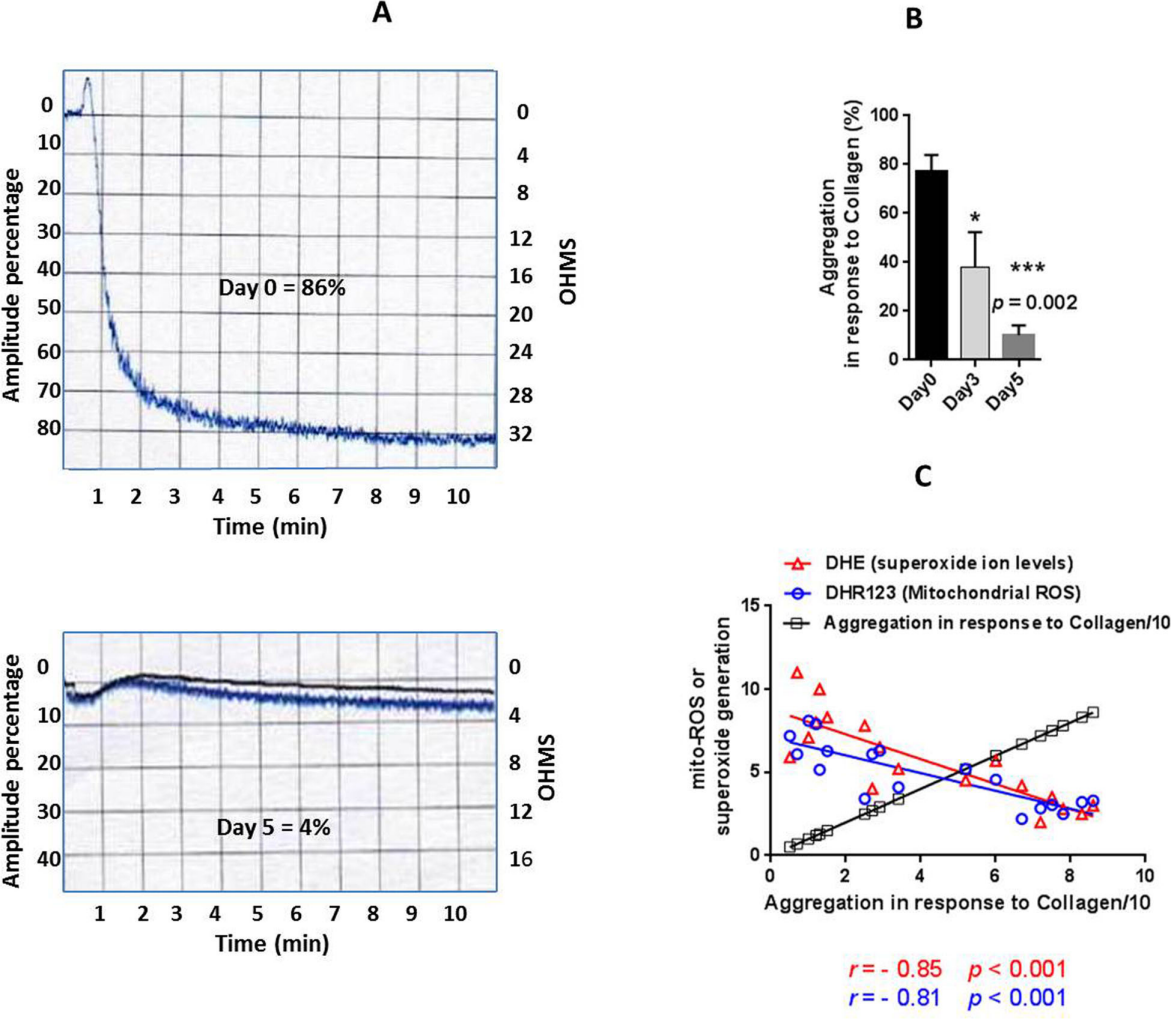
Collagen induced platelet aggregation and its correlation with ROS generation in stored platelets. **a** shows the representative images of platelet aggregation curve in response to collagen on day0 and 5 of storage. Graph **b** demonstrates platelet aggregations during storage. A direct correlation was found between collagen-dependent platelet aggregation and either mitochondrial ROS (*r =* − 0.81; *p <* 0.001) or cytosolic superoxide generation (*r =* − 0.85; *p <* 0.001) (graph **c**). Correlations were analyzed by Spearman’s rank correlation test. P values of less than 0.05 were considered to be significant. Correlation graphs have been plotted using accumulating data obtained during storage from day 0 to day 5. ***p < 0.001, **p < 0.01, *p < 0.05, ns: not significant *p* > 0.05 (*n =* 6)

Figure 5a demonstrates the representative images of platelet aggregation curve in response to collagen on day0 and 5 of storage. Platelet aggregation attenuated during storage with a significant drop started from day 3 (*p* < 0.05) reaching to the lowest levels (*p* = 0.002) in day 5 of storage (Fig. 4b). With further evaluation (Fig. 4c), a direct correlation was found between collagen-dependent platelet aggregation and either mitochondrial ROS (*r* = − 0.81; *p* < 0.001) or cytosolic superoxide generation (*r* = − 0.85; *p* < 0.001).

## Discussion

Generally, deleterious changes, so-called as platelet storage lesion (PSL) affects the quality and effectiveness of therapeutic platelets. The storage of PCs is associated with progressive platelet activation characterized by accumulating intra-platelet ROS, adhesive receptors shedding, platelet pro-coagulant activity and granule release leading to potential pro-inflammatory function of platelets [34–36]. Pro-coagulant activity (monitored by PS exposure) is also associated with apoptotic events, including cytochrome C release, caspase 3 activation, and the loss of mitochondrial respiration capacity as specific markers of apoptosis, however the significant rises of these markers are usually detected in long-stored platelets, from day 5 or 7 of storage [25]. Altogether, these are the stored-dependent changes of platelet which not only affect post-transfusion platelet survival but it may also attenuate platelet functional activity and effectiveness in circulation, while of note, some of these changes such as receptor shedding and the induction of pro-inflammatory or pro-coagulant phenotypes of platelets sound to be irreversible. Our previous studies on PCs indicated increasing levels of intra-platelet ROS during storage with the highest levels demonstrated in 5 days-stored platelets. We also showed that platelet storage can increasingly induce GPVI shedding [1]. Therefore, in this study given the key role of ROS in receptors modulation, we tried to evaluate storage-dependent correlation of ROS generation with GPVI modulation and platelet spreading on collagen matrix. Considering different sources for intra-platelet ROS, the levels of either superoxide or mitochondrial ROS in stored platelets have been first evaluated here. For the baseline study, the lowest levels of ROS generation were detected in freshly prepared PCs (0 day-stored platelets) whereas these platelets showed an influx of both superoxide and mitochondrial ROS generation in response to PMA and CCCP respectively. The phorbol ester, PMA that activates protein kinase C (PKC), is an important agonist which significantly induces NOX activity in platelets [37, 38] while as an uncoupler compound, CCCP induces mitochondrial lesion and apoptosis in platelets. CCCP is a protonophore which renders the mitochondrial inner membrane permeability to protons. This decreases proton gradient across the inner mitochondrial membrane and uncouples phosphorylation from oxidation while disrupting mitochondrial ATP synthesis. Therefore, CCCP treatment per se renders mitochondrial transmembrane potential (ΔΨm) and promotes Bax translocation to the mitochondria, leading to the release of apoptotic factors into the cytosol [39]. On the other hand, CCCP augments mitochondrial ROS generation [40, 41]. This elevated ROS can oxidize a main component of the inner mitochondrial membrane, cardiolipin and induce apoptosis through ΔΨm depolarization, mitochondrial Bax translocation, cytochrome C release, caspase-3 activation and PS exposure [42, 43]. However, given the fact that ROS scavenger, NAC can inhibit CCCP-induced reduction of ΔΨm and Bax translocation, presumably here ROS generation precedes the loss of mitochondrial transmembrane potential [39]. CCCP also showed to significantly promote GPVI shedding by ADAM17 rather than ADAM10 which is considered to be the specific sheddase for GPVI. Given the fact that ADAM 17 activity is modulated by ROS generation [1], the CCCP induced shedding might be also affected by oxidant pathways. Based on these background studies here, for the first time we showed CCCP-induced ROS generation in platelets that makes a direct link between platelet mitochondrial lesion and induced oxidative stress in an experimental setup. We also applied specific probe, DHR123 to detect mitochondrial-originated source of ROS in platelets [27]. In this study, a three-fold higher level of superoxide has been detected in 5 days-stored platelets. Intriguingly, this was even significantly higher than that induced by PMA as a potent NOX stimulating agent. Given this data, it seems that abovementioned pathways associated with mitochondrial lesion can be also involved in ROS generation here. To examine this theory, using DHR123, the similar patterns of mitochondrial ROS generation were also evaluated in stored platelets. Results showed 2.5 fold higher levels of ROS generation in 5 days-stored platelets at the levels comparable to that induced by CCCP which triggers serious mitochondrial damage and platelet apoptosis [44] . Conclusively although, some other research also reported the increasing levels of ROS generation during platelet storage [13, 14], our findings first highlighted the fact that ROS accumulation in 5 days-stored platelets is comparable to the highest levels of ROS generation experimentally induced with either potent non-physiological activator of NOX or mitochondrial damaging compound. In addition our results suggest a critical role for aged-dependent mitochondrial lesion of platelets in the augmentation of ROS elevation in longer stored platelets. This may indicate the significance of platelet lesion during storage that raises question about the quality of 5 days-stored PCs. The oxidant stress is generally indicated to be associated with different arrays of cellular damage and dysfunction. Augmented levels of ROS have been shown to disturb either natural proteomic or genomic materials in the cells [45, 46]. Several line of evidence indicated that a well-tuned physiologic concentration of ROS can act as an important modulator of platelets adhesive capacity. However, as a general rule, the oxidant stress induced by unleashed accumulation of ROS in platelets can seriously affect adhesion receptors shedding and their function [1]. We already showed the increasing levels of GPVI shedding in stored platelets with highest levels detected in the 5 days of storage [11]. Studies indicated an intense experimental shedding of GPVI induced by mitochondrial-targeting reagent CCCP that mimics platelet aging. PMA also activates protein kinase C (PKC) which triggers downstream ADAMs and significantly induces the shedding of platelet adhesion receptors including GPVI [47–49]. Results presented here also showed significant shedding of GPVI in fresh PCs treated with either PMA or CCCP while more interestingly, 5 days storage of platelets induces two folds higher level of GPVI shedding compared to that induced by PMA. GPVI shedding in 5 days-stored platelets was also statistically comparable to what induced by CCCP. This may indicate the levels of stored-dependent damage that affect platelets. In addition, the much higher shedding level in 5 days-stored platelets compared to that induced by PMA suggests the significant role of mitochondrial lesion rather than protein kinase- dependent pathways in these events. So far, several studies using different methods have indicated platelet adhesion loss to collagen during storage. In addition, we already showed that total adhesion of platelets to collagen is reversely correlated to GPVI shedding [11]. However, it seems that the evaluation of platelet spreading on collagen provides more information about the platelet signaling competence and functional quality beyond that seen for the simple adhesion [50]. In addition, since platelet firm adhesion and spreading is mainly mediated by GPVI receptor [1], platelets adhesion area seems to be a better indicator of GPVI competence rather than simple adhesion which might be also modulated by other less important receptors or be affected by artifacts. As presented here, in 5 days-stored PCs, the reduction of platelet spreading on collagen (calculated by platelet surface area) is much more significant than simple adhesion loss during storage. Such a decline in platelet adhesion surface area indicates that platelets profoundly lose their functional quality during long storage. On the other hand, platelet adhesion area correlates with GPVI shedding with the significance much higher than that was observed in simple adhesion. This may also suggest that platelet spreading on collagen is extremely GPVI-dependent. Notably, unlike our previous research [11], in current study we also evaluated the relevance of GPVI expression and adhesion capacities of platelet during storage. In general, GPVI shedding of stored platelets can be a more sensitive indicator of platelet activation than its expression, as to some extent the loss of GPVI expression by shedding might be compensated by platelet receptors re-expression and their dimerization due to continuous activating signals during storage [51, 52]. However, if the aim of GPVI evaluation is to examine platelet adhesive function, GPVI expression seems to be a better choice. Intriguingly here unlink shedding, while GPVI expression was directly relevant to platelet surface adhesion area (spreading), it did not show a significant correlation with simple adhesion. This confirms the fact that platelet firm adhesion is mainly modulated with GPVI expression while the earlier staged of adhesion to platelets (stable or unstable simple adhesion) might be orchestrated by other collagen receptors such as integrin α_2_β_1_ [2] or be due to nonspecific binding. Of note, whereas CCCP reduced GPVI expression to the levels which was comparable with those of 5 days-stored platelet, as an important shedding inducer, PMA did not show any significant effects. Two reasons might be considered to explain this event. First reason can be a lower effect of PMA on GPVI shedding compared to CCCP, which cannot overcome GPVI re-expression. The second one is PMA-induced GPVI dimerization that may compensate PMA-induced ADAM10 activity in GPVI shedding [52, 53]. Considering direct relevance between GPVI expression/shedding and ROS generation, we also evaluated any correlation of platelet adhesive capacity with ROS in stored platelets. Our data indicated that superoxide accumulation in PCs reversely correlates with both platelet adhesion number and spreading on collagen. Nonetheless, mitochondrial generated ROS was only relevant to platelet spreading on collagen while had no significant correlation with the number of adhered platelets. This finding suggests the key role of superoxide in earlier stage of platelet adhesion to collagen. This may also confirm other studies that have already highlighted the critical role of superoxide-induced oxidation of Cys residue (an unpaired thiol) located on the cytoplasmic tail of receptor, which results in rapid disulfide-dependent homodimerization of GPVI, igniting platelet adhesion to collagen [19, 20]. Collagen-induced platelet aggregation is also considered as another relevant functional assay which has shown to be reversely correlated with GPVI shedding [11]. PSL seriously affects collagen-induced platelet aggregation which significantly correlated with ROS generation. The significant reverse correlation of ROS with this observed platelet aggregation also highlights the potential involvement of stored-dependent oxidant stress in abnormal aggregation and thrombus growth.

## Conclusion

So far, several lines of evidence have indicated that platelet storage is associated with increasing levels of ROS. However, compared with potent ROS inducers, to what extent these molecules are accumulated in stored PCs had not been exactly described. Here for the first time, we demonstrated that the levels of ROS accumulation in 5 days-stored platelets are comparable to those induced by potent non-physiologic stimuli. These comparable effects on ROS inductions provide a verdict indicating the importance of oxidant stress in long stored platelets. It was plausible that such a level of oxidant stress can seriously affect platelet functional competence as for the first time we also showed a tremendous decrease in platelet spreading on collagen in 5 days-stored platelets associated with either the highest levels of GPVI shedding or its lowest expression (compared to those induced by CCCP as a toxic apoptotic agent). Taken together, the direct relevance of accumulating ROS with the significant loss of collagen-dependent platelet spreading/adhesion and aggregation was a striking sign of an oxidant-induced lesion that may raise serious question about the post-transfusion quality and competence of long-stored platelet products.

## Supplementary information

**Additional file 1.**

**Additional file 2.**

## Data Availability

The corresponding author can make available some dataset upon reasonable request.

## Abbreviations

ADAM: A disintegrin and metalloproteinase
CCCP: Carbonyl cyanide 3-chlorophenylhydrazone
CLEC-2: C-type lectin-like receptor 2
CPD: Citrate phosphate dextrose
Cys residue: Cysteine residue
DHE: Dihydroethidium
DHR: Dihydrorhodamine
DIOC6: 3,3′-dihexyloxacarbocyanine iodide
GPIbα: Glycoprotein Ibα
GPVI: Glycoprotein VI
MAPK: Mitogen-activated protein kinase
MTP: Mitochondrial transmembrane potential
NAC: N-acetylcysteine
NOX: NADPH oxidase
PC: Platelet concentrate
PKC: Protein kinase C
PMA: Phorbol-myristate-acetat
PPP: Platelet-Poor Plasma
PRP: Platelet-rich plasma
PS: Phosphatidylserine
PSL: Platelet storage lesion
ROS: Reactive Oxygen Species
vWF: Von Willebrand factor
