# Supplementary material for: Collagen-dependent platelet dysfunction and its relevance to either mitochondrial ROS or cytosolic superoxide generation: a question about the quality and functional competence of long-stored platelets
Source: Thromb J. 2020 Aug 31;18:18. doi: 10.1186/s12959-020-00233-y (PMC7457792; doi:10.1186/s12959-020-00233-y)
Supplement: Supplementary file 1 — Additional file 1. [file 12959_2020_233_MOESM1_ESM.docx]

**Supplementary Method**

**Reagents**

Mouse IgG1 κ Isotype controls (FITC conjugated) were from Miltenyi Biotec (Germany). FITC mouse anti human GPVI was purchased from Santa Cruz Biotechnology. Monoclonal antibodies against human GPVI (clone HY101) as well as anti-mouse IgG1 HRP were from eBiosciences Inc (USA). Dihydroethidium was purchased from Cayman Chemical (DHE; Cayman Chemical, AnnArbor, MI, USA). Dihydrorhodamine (DHR) 123 and other reagents and chemicals were from Sigma Aldrich (USA).

**QC parameters of PCs**

Assessment of total volume, PLT count (per unit), WBC count (per unit) were carried out on PCs after preparation and during storage at each time points. PLT count and WBC count were measured using a hematology full blood analyzer (XE-2100, Sysmex, Milton Keynes, UK).

**Analysis of intra-platelet ROS generation**

Dihydrorhodamine (DHR)123 is a permeable mitochondrial-avid component that can undergo oxidation to the fluorophore rhodamine 123 [[1](#_ENREF_1)]. The oxidized rhodamine has a tendency to remain within the mitochondria after tautomerization of its equivalent amino groups. DHR provides the advantage of being oxidized by both H_2_O_2_ and O_2_^--^ while emitting a strong fluorescence signal, this makes it a very sensitive indicator for detection of ROS generation in mitochondria [[2](#_ENREF_2)]. To analyze cytosolic production of O_2_^--^, dihydroethidium has also been used. To perform the assays, platelets (2×10^7^/ml) were incubated with either 1 μM DHR 123 or 5 μM dihydroethidium for 30 min at 37ºC, in the presence and absence of either 100 μM CCCP (carbonyl cyanide m-chlorophenylhydrazone) or 2 μM PMA (phorbol 12-myristate13-acetate) respectively (both compounds pre-incubated with platelets for 30 minutes) . Cells were then subjected to a flow cytometer (CyFlowSpace, Partec GmbH, and Germany) where a total of 20,000 PLT events were acquired. The flow cytometric settings were optimized for the acquisition of platelets by logarithmic signal amplification in all 3 detectors (forward and side scatter channels and fluorescence channels FL1/FL2). For analysis, the gate was set around intact PLT population (R1) as defined by forward and side scatter characteristics and confirmed by the presence of platelets expressing either CD61 or GPIbα (in a parallel experiment). The mean fluorescence of platelets expressing DHR 123/DHE in comparison with the background (negative control) was recorded. Data were analyzed with FLOWJO software (Tree Star Inc, OR, USA). Supplementary figure1 has schematically demonstrated the gating strategy and analysis protocol with more detail.

**Western blotting analysis of GPVI shedding**

Platelet poor plasma (PPP) obtained from 6 PRP-PCs with the platelet count of 5×10^8^ /ml were subjected to two steps ultracentrifugation (2×10^4^g for 30 minutes each time) and microparticles (MPs)-free supernatant was separated and subjected to western blot analysis. To examine shedding responses to stimuli, platelets (5×10^8^ /ml) were incubated with either 100 μM CCCP (carbonyl cyanide m-chlorophenylhydrazone) or 10 μM PMA (phorbol 12-myristate13-acetate) for 30 minutes prior to fractionation. Microparticle-free samples were subjected to SDS-PAGE and western blotting analysis to identify soluble GPVI. To prevent interfere with the proteins bands, albumin was fractionated from samples before experiments [[3](#_ENREF_3)]. Proteins were then Immunoblotted using primary specific monoclonal antibody, HRP-conjugated secondary antibody and enhanced chemiluminescence reagent. Finally, bands were visualized by ChemiDoc XRS+ system using image Lab software (Bio-Rad Laboratories, inc. USA). For each run of shedding analysis, the free-MPs supernatant of samples obtained from PRP-PCs (with the same count of platelets) were subjected to western blot analysis along with a positive control of shedding. For each run, the intensities of samples were calculated in order to [semi-quantitative](https://www.google.com/url?sa=t&rct=j&q=&esrc=s&source=web&cd=1&cad=rja&uact=8&ved=0CBwQFjAAahUKEwiUitu7kZfJAhXDvhQKHcaABXc&url=http%3A%2F%2Fwww.merriam-webster.com%2Fdictionary%2Fsemiquantitative&usg=AFQjCNFUOTYtf7jD_QHzmaUhy46VDCXkvw&bvm=bv.107467506,d.bGg)ly analyze and compare the amounts of shed receptors from day 1 to day 5 of storage.

**Flow cytometery analysis of GPVI expression**

Washed platelets obtained from 6 PRP-PCs were re-suspended in Tyrode buffer with count adjusted to 2×10^7^/ml. Platelets were incubated with FITC mouse anti human GPVI for 30 minutes at 37ºC, in the presence and absence of either 100μM CCCP (carbonyl cyanide m-chlorophenylhydrazone) or 2μM PMA (phorbol 12-myristate13-acetate) respectively (both compounds pre-incubated with platelets for 30 minutes). Platelets were stained with anti-GPVI for 30 min. Isotype-matched control antibodies were used to determine background staining created by non-specific binding. Cells were then fixed in 1% paraformaldehyde in PBS and subjected to flow cytometer (CyFlow®Space, Partec GmbH, Germany) where a total of 20,000 platelet events were acquired. The flow cytometer settings were optimized for the acquisition of platelets by logarithmic signal amplification in all four detectors (forward and side scatter channels and fluorescence channels FL1 and FL2). For analysis, the gate was set around intact platelet population as defined by forward and side scatter characteristics and GPVI expression was quantified as the mean fluorescence intensity (MFI). Data were analyzed with FLOWJO software (Tree Star Inc., OR, USA).

**Bacterial cultures**

Bacterial cultures were performed on day 5 for each bag of platelets, including both aerobic and anaerobic cultures

**Statistical analysis**

For comparison of the parameters in different days, data were analyzed by Kruskal-Wallis test with Dunn’s multiple comparison test. Mann–Whitney U test was also applied to compare parameters between two groups (platelets treated with different agents compared to control). Correlations were analyzed by Spearman's rank correlation test. P values of less than 0.05 were considered to be significant, using GraphPad Prism software (GraphPad Prism Software, Inc., San Diego, CA).

**References:**

[1] G. Rothe, A. Emmendorffer, A. Oser, J. Roesler, G. Valet, Flow cytometric measurement of the respiratory burst activity of phagocytes using dihydrorhodamine 123, J Immunol Methods 138(1) (1991) 133-5.

[2] J. Yee, B. Giannias, B. Kapadia, L. Chartrand, N.V. Christou, Exudative neutrophils. Modulation of microbicidal function in the inflammatory microenvironment, Arch Surg 129(1) (1994) 99-105.

[3] Q. Fu, C.P. Garnham, S.T. Elliott, D.E. Bovenkamp, J.E. Van Eyk, A robust, streamlined, and reproducible method for proteomic analysis of serum by delipidation, albumin and IgG depletion, and two-dimensional gel electrophoresis, Proteomics 5(10) (2005) 2656-64.
