# Supplementary figures and images for: Collagen-dependent platelet dysfunction and its relevance to either mitochondrial ROS or cytosolic superoxide generation: a question about the quality and functional competence of long-stored platelets
Source: Thromb J. 2020 Aug 31;18:18. doi: 10.1186/s12959-020-00233-y (PMC7457792; doi:10.1186/s12959-020-00233-y)

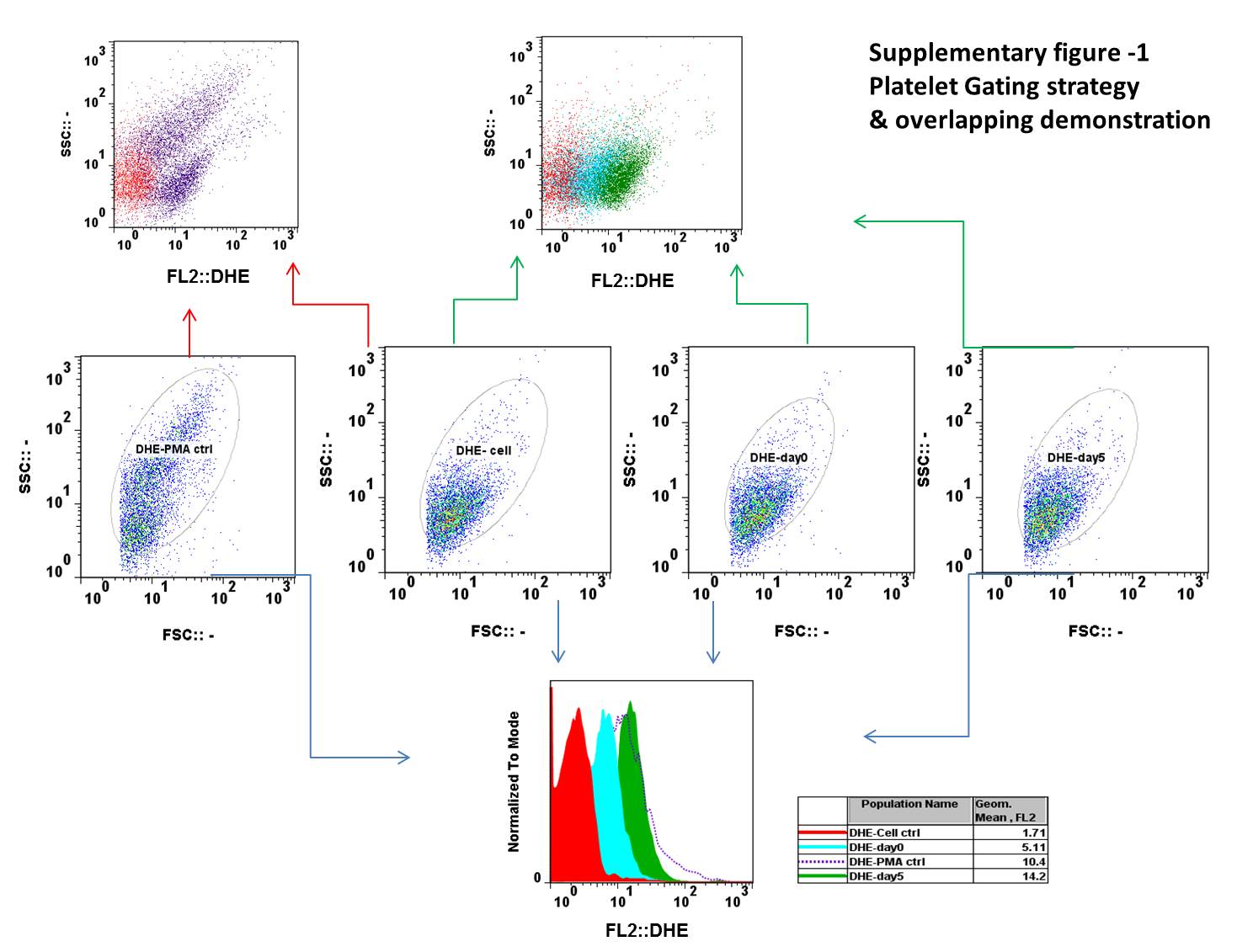

Supplement: Supplementary file 2 — Additional file 2. [file 12959_2020_233_MOESM2_ESM.jpg]
